# CLIMATE-CREATIVE COMMUNITIES TO PROMOTE PHYSICAL ACTIVITY AND HEALTH EQUITY IN LOW-INCOME SETTINGS

**DOI:** 10.1093/geroni/igae098.0858

**Published:** 2024-12-31

**Authors:** Ladda Thiamwong, Dahee Kim, Rui Xie, Christopher Emrich, YunYing Zhong, Jennifer Crook, Timothy Hawthorne

**Affiliations:** University of Central Florida, Orlando, Florida, United States; University of Central Florida, Orlando, Florida, United States; University of Central Florida, Orlando, Florida, United States; University of Central Florida, Orlando, Florida, United States; University of Central Florida, Orlando, Florida, United States; University of Central Florida, Orlando, Florida, United States; University of Central Florida, Orlando, Florida, United States

## Abstract

Extreme heat is one of the most fatal weather hazards, and limited data suggest that low-income older adults (LOAs) who are exposed to extreme heat are less likely to participate in physical activity (PA). To develop climate-creative communities, we conducted onsite survey interviews with LOAs (n=41) and a focus group with community-based organizations (CBOs) (n=21). LOAs tended to have less frequent moderate to vigorous PA and had higher numbers of falls in summer compared to fall; 71% felt uncomfortable with extreme heat, and 69% rated their thermal sensation as slightly warm to very hot on extreme heat days. Regarding self-management on hot days, 89% drank water, 13% drank juice/favored beverage, 2% drank alcohol, 29% stayed inside, and 22% reduced PA. More than 50% stated heat or hot weather impacted their sleep, food consumption, and hydration; 32% reported heat impacted social connections, and 47% had no access to community resources (CR). The top five barriers to accessing CR were health issues (20%), don’t have time (11%), transportation from home to CR (9%), lack of interest (8.9%), and lack of knowledge and information about the services (6.7%). A focus group with CBOs (n= 21) agreed that there is currently scarce communication/coordination of relevant CR, and multilateral efforts are urgently needed to co-create community-based, culturally appropriate communication and outreach programs to encourage LOAs’ participation in PA, engage them in discussions about heat-health issues and provide them with access to shared CR to support their built community’s resilience in adapting to rising temperatures.

